# PD-1/PDL-1 Inhibitors and Cardiotoxicity; Molecular, Etiological and Management Outlines

**DOI:** 10.1016/j.jare.2020.09.006

**Published:** 2020-10-03

**Authors:** Mohammed Safi, Hyat Ahmed, Mahmoud Al-Azab, Yun-long Xia, Xiu Shan, Mohammed Al-radhi, Abdullah Al-danakh, Abdullah Shopit, Jiwei Liu

**Affiliations:** a Department of Oncology, First Affiliated Hospital of Dalian Medical University, Zhongshan Road No. 222, Dalian 116021, China; b Department of Stomatology, Oral Pathology, Dalian Medical University, Zhongshan Road No. 222, Dalian 116021, China; c Department of Immunology, Guangzhou Institute of Pediatrics, Guangzhou Women and Children's Medical Center, Guangzhou Medical University, Guangzhou, 510623, China; d Head of Department of Cardiology, Vice president of the First Affiliated Hospital of Dalian Medical University, 222 Zhongshan Road, Dalian 116021, Liaoning, China; e First Affiliated Hospital of Dalian Medical University, Zhongshan Road No. 222, Dalian 116021, China; f Department of Urology, Second Affiliated Hospital of Dalian Medical University, Zhongshan Road No. 222, Dalian 116021, China; g Department of Urology, First Affiliated Hospital of Dalian Medical University, Zhongshan Road No. 222, Dalian 116021, China; h Department of Pharmacology, Dalian Medical University, Zhongshan Road No. 222, Dalian 116021, China; i Head of Department of Oncology First Affiliated Hospital of Dalian Medical University, Zhongshan Road Dalian, Dalian Liaoning Province 116044, China

**Keywords:** PD-1, PDL-1, Cardiotoxicity, Myocarditis, Heart block

## Abstract

**Background:**

The US Food and Drug Administration (FDA) has approved several immunotherapeutic drugs for cancer since 2010, and many more are still being evaluated in other clinical studies. These inhibitors significantly increase response rates and result in the treatment of patients with advanced cancer. However, cancer immunotherapy leads to essential cardiac toxicity properties that have become distinct from other cancer patients’ care and are mostly related to their etiology.

**Aim of review:**

As potential implications, the occurrence of cardiovascular adverse events is particularly challenging and needs a comprehensive understanding of overall cancer-related etiology, clinical outcomes with different variable severity, and management.

**Key scientific concepts of review:**

In terms of improving the overall survival of patients with cancer, clinicians should be careful in selecting either programmed cell death-1 (PD-1) or its programmed cell death ligand (PDL-1) inhibitors by evaluating their risk and clinical benefit for early intervention and decrease the level of morbidity and mortality of their patients. This review focuses on the effectiveness of PD-1/PL-1 antibodies and associated cardiotoxicity adverse events, including etiological mechanisms, diagnosis, and treatment.

## Graphical abstract

**Figure:**
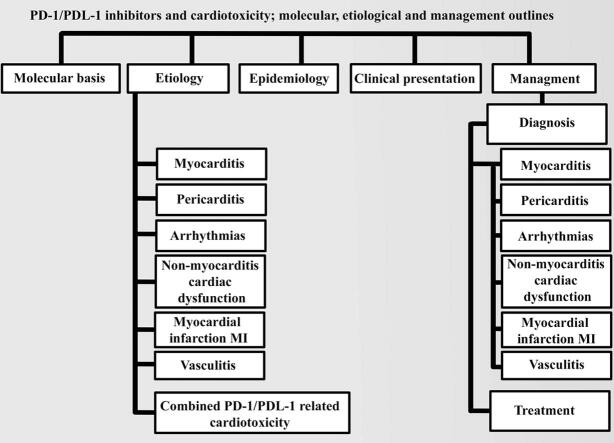

## Introduction

PD-1 is a protein identified on T-cells, and when bound to another protein called PDL-1, it prevents the killing of other cells by T-cells. Based on this mechanism, many of the inhibitors for PD-1/PDL-1 proteins have been launched to upregulate T-cells’ ability to fight cancer cells and increase survival. In practice, PD-1/PDL-1 inhibitors have characteristically different mechanisms from conventional chemotherapy; however, this generates a wide range of immune-related adverse events (irAEs) in all body tissues. irAEs affect significant body organs that can lead to life-threatening toxicities. Recently, immune check inhibitors have been approved, and treatment options in clinical practice have been extended [1]. These drugs include nivolumab, pembrolizumab, and cemiplimab as PD-1 inhibitors for metastatic melanoma and non-small cell lung cancer (NSCLC) treatment; however, the latter is explicitly approved for advanced cutaneous squamous cell carcinoma [2]. Atezolizumab, durvalumab, and avelumab are three FDA-licensed PDL-1 inhibitors for more than ten cancer types, including melanoma, NSCLC, head and neck squamous cell carcinoma, esophageal cancer, gastric cancer, kidney cancer, bladder cancer, cervical cancer, Hodgkin’s and non-Hodgkin’s lymphoma, Merkel cell carcinoma, and colorectal cancer. Furthermore, a new anti-PDL-1 inhibitor (envafolimab) has started phase 1 trials in the US and Japan and a phase 2 registration trial in China with MSI-H tumor patients or in combination as a phase 3 registration trial in cholangiocarcinoma patients [3].

Improvements in therapeutic effectiveness must be tested against potentially unsafe incidents when considering the care strategies for PD-1/PDL-1 monoclonal antibodies, and each adverse outcome must be accordingly assessed separately. In a systematic analysis of safety on general treatment-related adverse events (trAEs) and irAEs among different types of PD-1/PDL-1 inhibitor-related therapeutic regimens concurrently, anti-PDL-1 monotherapy had excellent safety. By contrast, anti-PD-1 drugs had worse and damaging effects [4]. Nevertheless, further studies are needed to find a thorough risk and etiological model for identifying pathways that result in toxicity and to enhance further the recent approaches to early detection and treatment [5], [6]. Cardiotoxicity is the most feared and undesirable toxicity that can arise after PD-1/PDL-1 drug administration that is still underreported and/or underestimated, and specific definitions and guidelines for controlling it are yet to be formulated [7].

While PD-1/PDL-1 inhibitors are periodically approved and expanded for use by the FDA, there is still little evidence in the literature concerning the possible differences between PD-1 and PDL-1 inhibitors and related cardiotoxicity that can improve the best choice of specific monoclonal antibodies. Our review will outline the etiology, diagnosis, and cardiovascular toxicity management of PD-1/PDL-1 drug therapy in depth.

## Molecular basis

In 1992, Tasuku and colleagues conducted the first research on PD-1 and discovered its upregulation during programmed cell death [8]. Later, no direct relationship was found with apoptosis, but negative T-cell regulation and immune actions were mediated by T-cells. In preclinical studies, deficient PD-1 upregulation tended to result in many autoimmune diseases [9].

Two hundred eighty-eight amino acids are composed of a PD-1 protein that is frequently expressed in many types of cells, including activated T-cells, B-cells, monocytes, natural killer cells, dendritic cells (DCs), CD4+ cells, and CD8+ cells [10], [11], [12]. In T-cells, the expression of PD-1 may be regulated by IL-2, IL-7, IL-15, and IL-21 receptors and by various T-cell regulators [13], [14], [15], [16]. A strongly expressed PD-1 in Treg cells contributes to their development and work by boosting the expression of Foxp3 (forkhead box P3), a crucial transcription factor of the Treg cell population [17]. Thus, the main action of PD-1 protein signaling is to facilitate the inhibition of T-cell receptors by direct action or indirect blockade of signaling cascades co-stimulated by receptors.

In the literature so far, PD-1 ligands have been identified and described as PDL-1 (B7-H1, CD274) and PDL-2 (B7-DC, CD273) with markedly different cellular distribution profiles at the amino acid level with 38% homology [18]. PDL-1 was first recorded in 1999 on hematological (monocytes, macrophages, DCs, T-cells, and B-cells) and some non-hematopoietic cell populations by Chen and colleagues through the sequence of CD80 and CD86 [19]. Once activated, it is upregulated on macrophages and monocytes. PDL-2 is mostly expressed on DCs [20]. During most autoreactive T-cell deletion, the thymus plays a central tolerance mechanism in healthy tissues, including the heart, as it regulates many cells [21], [22]. PD-1 and PDL-1 proteins lead to immunotolerance and prevent immune reactions to cardiac antigens [23], [24], [25]. In preclinical studies, both proteins were prominently triggered in cardiac tissues, and deregulation results in dilated cardiomyopathy and life-threatening myocarditis [26].

Furthermore, the distinct interactions between PD-1 and PDL-1 result in remarkable immune regulation, and cardiac immune-mediated adverse effects following myocardial infarction (MI) and ischemia–reperfusion injury have also been identified in recent studies [27], [28].

## Etiology

In preclinical models, the deletion of PD-1 encoding genes may contribute to autoimmune myocarditis [27]. An early histological review of myocarditis in humans established the involvement of CD4 and CD8 cells and macrophages and suggested that it is the critical pathophysiological driver of the disease [29]. IgG deposition in PD-1^–/–^ mice was the baseline for inflammation that was also demonstrated by troponin 1 as a type of autoimmune myocarditis model. PDL-1 expression has cardioprotective effects by suppressing the inflammation process and direct cardioprotective signaling during acute ischemia and MI. Furthermore, the acquired heart problems mediated by systemic inflammation such as tumor necrosis factor-α and PD-1/PDL-1 inhibitors may exaggerate or accelerate the decompensation of pre-existing heart diseases (heart failure, arrhythmia, heart injury, and dysfunction) in susceptible individuals.

Therefore, PD-1/PDL-1 monoclonal antibodies can cause different heart problems through autoimmune T-cell-mediated myocarditis, and the direct inhibition of PDL-1 can accelerate pre-existing heart diseases via non-inflammatory cardiomyocyte dysfunction with or without evidence of immune response [30]. Although cardiotoxicity related to PD-1 and PDL-1 inhibitors has been underestimated, many recent studies provide the impression that its incidence is rare and early intervention decreases deadly side effects of the immune checkpoint blockade associated with a tissue injury [31]. Blocking of immune regulation by PD-1/PDL-1 inhibitors that are directed against PDL-1 or PD-1 (receptor on T-cell) may induce cells against tumor site cells and other tissues [32]. Carolyn et al. reported evidence of idiopathic fulminant lymphocytic myocarditis and coagulative necrosis with the absence of PDL-1 expression [33].

Okazaki et al. found an increased immunoglobulin titer against cardiac troponin I, an antibody contributing to hypertrophic cardiomyopathy etiology [34]. Coadministration of ipilimumab and nivolumab in cynomolgus monkeys at levels higher than those used in clinical practice has led to widespread lymphocytic invasion in various tissues, including the dominant involvement of T-cells in the myocardium. Some of these models have shown histopathological signs of cardiomyocyte necrosis, accompanied by an increase in cardiac serum biomarkers. Transcriptomic results revealed the elevated production of chemokine receptors in infected monkey heart tissues, indicating a more significant movement of activated T-cells [30], [35]. Furthermore, PDL-1 plays a crucial role in reducing cardiac inflammation following infection by displaying elevated distribution of IFNπ, FasL, CD40, perforin, and viral genomes in myocardial tissue in the presence of PDL-1 blocking antibodies [36]. In large mice (Murphy Roths), the near absence of PDL-1 contributes to fatal myocarditis and accumulation of macrophages and T-cells in the whole heart along with cardiac-specific autoantibodies [25]. Hence, those with PDL-1 positive tumors treated with PDL-1 inhibitor had a higher objective response rate (43%) than in those with PDL-1 negative tumors (11%) [37]. Accordingly, in an expanded clinical trial conducted in patients with different types of cancer treated with monoclonal antibody anti-PDL-1, the best tumor response was seen in those with high levels of PDL-1, which could help in making decisions regarding immunotherapy treatment [20], [38] Table 1.

**Table 1:** Cardiotoxicity at time of FDA approval.

Drug	Approval	Participants	Antibody	Cancer type	Cardiotoxicity - related events^a^
Pembrolizumab	2014	834 patients who had no more than one line of prior systemic therapy and not received ipilimumab	Humanized anti-PD-1 IgG4	Melanoma Non-small cell lung carcinoma Squamous cell carcinoma of head and neck Urothelial carcinoma, Head, neck squamous cell cancer	Various heart diseases; 0–4%*: -Myocarditis (0.5%)-Myocardial infarction (2%)-Pericarditis (2%)-Arrhythmia (4%)-Takotsubo syndrome
Nivolumab	2014	142 patients, stratified by BRAF V600 mutation status, 109 patients with BRAF V600 wild–type melanoma	Human anti-PD-1 IgG4	Classical Hodgkin lymphoma Melanoma Non-small cell lung cancer Renal cell carcinoma Small cell lung cancer Squamous cell carcinoma of head and neck	-Myocarditis(<1%)**-Ventricular arrhythmia (1–10%)-Pericarditis (<1%)
Atezolizumab	2016	Urothelial carcinoma; 310 patients had disease progression during or following a platinum platinum–containing chemotherapy, 32% have PD–L1expression	Humanized anti-PDL-1 IgG1	Non-small cell lung cancer, urothelial carcinoma	- Myocarditis** (<1%)-Myocardial infarction
Durvalumab	2017	Urothelial carcinoma; 182 patients who progressed on platinum–containing chemotherapy	Human anti-PDL-1 IgG1	Non-small cell lung cancer, urothelial carcinoma	Myocarditis < 1%
Avelumab	2017		Human anti-PDL-1 IgG1	Urothelial carcinoma, Merkel cell carcinoma	Myocarditis < 1%
Cemiplimab	2018	Efficacy reported in 250 patients; Phase II Trial	Human anti-PD-1 IgG4	Cutaneous Squamous cell carcinoma	Myocarditis

a Label of Food and Drug Administration FDA.

* Included in the label of Food and Drug Administration (Keynote-087- Keynote-170- Keynote-006).

** Included in the label of Food and Drug Administration (CHECKMATE-037-clinical trials of OPDIVO administered as a single agent or in combination with ipilimumab and POPLAR trial of atezolizumab).

**Fig. 1:**
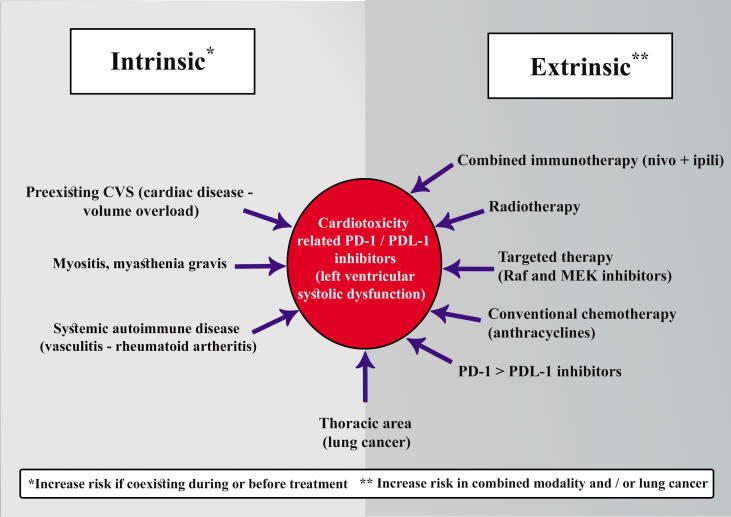
Intrinsic and extrinsic etiological factors for cardiotoxicity-related PD-1/PDL-1 inhibitors.

However, one study suggests that volume overload may increase irAEs and thus organ dysfunction in a population with multiple comorbid illnesses and heavily pretreated population. Nevertheless, no clear reported pathways of anti-PD-1/PDL-1 inhibitors as extrinsic factors with organ dysfunction as intrinsic ones have said so far [39] Fig. 1

### Myocarditis

Preclinical studies on myocarditis and heart failure have revealed the pivotal role of PD-1/PDL-1 signaling pathways that lead to cardiac autoimmune reaction [40], [41]. The negative effect of the progression of cardiac side effects has been demonstrated in preclinical studies [42]. In mice, knocking out PD-1 increased the mortality rate [43].

Post-mortal fatal immune inhibitors revealed confirmed myocarditis with significant inflammatory cell infiltration, increased extracellular space, and loss of cardiomyocytes; however, it is still not well understood of their etiology and pathophysiology. In one literature review with a PD-1 inhibitor, nivolumab combined with ipilimumab confirmed that CD4+ and CD8+ cardiomyocytes infiltrate only in T-cells and macrophages [25], [29]. The T- cells from the heart and tumor or skeletal muscle were confirmed to have the same systemic T-cell clones and cardio-specific genes such as cardiac troponin 1 and myosin heavy chain seen by RNA sequencing. Cardiomyocytes can be activated by the tumor enhancing – T-cell population that leads to cross-reacts with myocardium antigens, especially after PD-1/PDL-1 inhibitors administration. However, pretreatment T-cell clones testing recommended getting a baseline and standard prediction [31], [44].

### Pericarditis

In combination or as monotherapy in the treatment of different cancer types (metastatic melanoma, metastatic renal cell carcinoma), anti-PD-1/PDL-1 drugs lead to pericarditis with an incidence of less than 1% (30). Many studies reported that anti-PD-1/PDL-1 drugs cause repeated pericardial and pleural effusions that may contribute to pericarditis [2]. Nevertheless, whether any of the cases of pericardial effusion related to anti-PD-1/PDL-1 inhibitors may have underlying pericarditis remains unknown. Many malignancies are associated with pericardial effusions, especially lung cancer, which is one of the most common cancers treated with immunotherapy. Consequently, the appearance of a new or progressing pericardial effusion may increase the possibility of anti-PD-1/PDL-1-related perimyocarditis but should not be regarded as a separate condition from other findings. In terms of histology, numerous studies indicated that immune checkpoint treatment is accompanied by acute fibrinous pericarditis with mixed inflammatory infiltrations in the pericardial wall and the deposition of surface fibrins [45].

### Arrhythmias

In a broad cohort review of hospitalized patients with myocarditis, 33.7% experienced arrhythmia (the most severe being ventricular tachycardia and atrial fibrillation) [46]. Moreover, the same forms of lethal arrhythmias and third-degree heart block have been recorded in patients receiving PD-1/PDL-1 inhibitors. The mechanism of myocarditis that gives rise to arrhythmia is still under debate. Different pathways such as myocarditis, pre-existing cardiovascular diseases, past cardiotoxic chemotherapy, uncommon myocardial metastases, old age, and systemic inflammatory conditions could contribute to arrhythmia in cancer patients treated with PD-1/PDL-1 inhibitors. As a result, the need to stratify the risk for the possibility of arrhythmia is highly recommended for early intervention.

### Non-myocarditis left ventricular dysfunction

#### Dilated cardiomyopathy

The most reported cases of left ventricular dysfunction induced by PD-1/PDL-1 inhibitors have been related to or secondary to myocarditis. However, there are cases in which myocarditis does not seem to be a relevant factor, and this is because of the (1) absence of cardiac troponin 1, (2) no evidence of cardiac toxicity (active inflammation) in cardiac PET/CT or MRI, and (3) no post-mortem inflammatory infiltrates on endomyocardial biopsy (EMB). The presence of dilated cardiopathy without edema (FDG PET/MRI) or with increased troponin markers during treatment with PD-1/PDL-1 inhibitors indicates the existence of non-inflammatory left ventricular dysfunction [47]. One interesting study reported that the reduction of PD-1 expression resulted in dilated cardiomyopathy with severely impaired contraction and congestive heart failure, which resulted in sudden death [45]. PDL-1 expression on cardiomyocytes is upregulated in cardiac diseases such as ischemia–reperfusion injury and left ventricular hypertrophy in preclinical studies [27].

#### Takotsubo syndrome

Takotsubo syndrome is another form of left ventricular dysfunction defined as a characteristic of stress cardiomyopathy. It is characterized by transient left ventricular dysfunction and ECG changes similar to those of acute myocardial infarction (MI). These changes are attributed to the direct effect of catecholamines on cardiomyocytes with different histological patterns of lesions called myocytolysis [48]. This syndrome was reported in a case report as a complication of PD-1 inhibitor (nivolumab) combined with anti-CTLA-4. Nevertheless, the occurrence of myocarditis has not been excluded [49], [50]. To date, there is no reported case with PD-1/PDL-1 inhibitors either as monotherapy or combination therapy.

### Myocardial infarction MI

Atherosclerosis is a common cardiovascular disease that usually develops as chronic, known, or unknown inflammation caused by many disorders. The leading cause of MI and thromboembolic stroke is a rupture of atherosclerotic plaque. Theoretically, the same will be in PD-1/PDL-1; however, the mechanism has not been studied. PD-1/PDL-1. As in FDA label, MI was reported in the trial of checkpoint inhibitor of atezolizumab. In addition, a meta-analysis of 22 clinical trials with single PD-1 or PDL-1 inhibitors in NSCLC (pembrolizumab, nivolumab, and atezolizumab) suggested a 5.2% overall rate of significant cardiac events during a brief follow-up period with 1.0% for fatal MI [51]. Nevertheless, highly considering the radiation and site of cancer were suggested to be incorporated in the cardiotoxicity. Additionally, a novel therapeutic approach for this fatal complication is required in order to decrease its incidence during immunotherapy [44], [52]. Although PD-1/PDL-1 inhibitors are indicated for a wide range of tumors, we still need to understand and confirm the exact relation of cardiac events with PD-1/PDL-1 inhibitors.

### Vasculitis

Most of the recorded trials showed that immunotherapy-related vasculitis, such as temporal arteritis and rheumatic polymyalgia, predominantly affect older (age 70–80) and white populations [53]. Several pieces of evidence suggest the substantial role of PD-1 pathways in vasculitis pathophysiology. In patients with different types of vasculitis, single-nucleotide polymorphisms in genes encoding PD-1 were correlated with T-cell hyperactivity at the vascular level [54]. Elevated concentrations of inflammatory markers promoting CD4 cells, macrophages, and multinucleated giant cells were located in medium to large arteries of patients with temporal arteritis, secondary to a decrease in PD-1 gene expression and transcription [55]. Thus, an immune system that favors the production of vasculitis is likely to be established in the setting of anti-PD-1 substance usage. In terms of histology, fibrous vasculitis with mixed inflammatory infiltrations in the vessel wall can be seen.

### PD-1/PDL-1 cardiotoxicity in multiple modality treatment

Many cancer patients have been under several potential modalities such as chemotherapy, radiotherapy, and cardiotoxic targeted therapy before using PD-1/PDL-1 inhibitors, which lead to amplified cardiotoxic effects and misdiagnosis like Rapidly Accelerated Fibrosarcoma kinase (Raf) and Mitogen-Activated Protein Kinase inhibitors (MEK), anthracyclines, and Vascular Endothelial Growth Factor (VEGF) tyrosine kinase inhibitors. In one trial of combined PDL-1 inhibitor with tyrosine kinase inhibitor for renal cell carcinoma, potential myocarditis was observed in one case (2%) of 50 patients [56]. Furthermore, anti-PD-1 blockade in a preclinical model amplified radiation cardiotoxicity, indicating the interaction between PD-1 inhibitors and radiation therapy [52]. In one case series, different cardiac diseases with varying grades of toxicity were reported in patients under PD-1 inhibitor (pembrolizumab) with or without cardiotoxic pretreatment (radiotherapy, immunotherapy, and targeted cancer drugs) [57]. The synergistic effect of the combinations of PD-1/PDL-1 or both with classical chemotherapy, targeted therapy, or radiation therapy should be considered during treatment and final diagnosis of cardiotoxicity.

## Epidemiology

Many clinical trials and meta-analyses reported that the incidence of cardiotoxicity symptoms is higher in patients with end-organ failure than in those without organ dysfunction. Over time, the WHO database recorded an increase in immunotherapy-induced myocarditis. This finding indicated that the incidence of cardiotoxicity effects is increasing because of the increased use of immune checkpoint inhibitors (ICIs) with improved recognition of ICI-induced myocarditis (0.7% for cardiac tamponade, 0.5% for myocarditis, 1.0% for MI, 1.0% for cardiac arrest) [58], [59]. Additionally, a meta-analysis of 112 trials involving 19,217 patients found general toxicity-related fatality of anti-PD-1 fatality rates 0.36% more than of anti-PDL-1 0.38% and cardiac events in 4 and 3 cases (from 12 and 25 cases) respectively [60].

In a large meta-analysis of 22 anti-PD-1/PDL-1 clinical trials, the incidence of cardiovascular events was more than that of the Bristol-Myers Squibb Pharmacovigilance Database with (2%) vs. (1%) respectively. However, the occurrence of each cardiac event was comparatively low (cardiorespiratory arrest 1%, heart failure with MI 1.0%, and stroke 2%) [44]. Cardiotoxicity related to PD-1/PDL-1 and CTL-4 inhibitors has been reported in 30 patients in a median of 65 days after an average of three infusions (range, 1–33). The majority of patients manifested left ventricular systolic dysfunction (79%) [61]. Atrial fibrillation, ventricular arrhythmia, conduction disorders, and Takotsubo syndrome-like appearance were observed in 30%, 27%, 17%, and 14% of patients, respectively. The associated signs of myositis were present in 23% of patients. Cardiovascular mortality (mostly ventricular arrhythmia) reported in (27%) that almost associated with conduction abnormalities (80%) in patients with the combination of either of anti-PD-1/PDL-1 an anti-CLTA-4 and severity of myocarditis was observed in 57% more than those with anti-PD-1/PDL-2 monotherapy [20], [58], [61].

Although cardiotoxicity has been reported in most trials and case series, the PACIFIC trial has not published any cardiac event in the treatment group (PDL-1 inhibitor durvalumab after concurrent chemotherapy and radiotherapy) [62]. Moreover, the pathophysiology of PD-1 or PDL-1 inhibitors should pay awareness of concurrent autoimmune disorders that comprise 42% (42 of 101 irAEs). Besides, it was reported conditions as severe myositis in 25 patients and myasthenia gravis in 11 patients [58], [59].

## Clinical presentation

In cardiac toxicity related to anti-PD-1/PDL-1 drugs, most patients show symptoms shortly after treatment [31]. Although a small percentage of toxicity was observed after several months [63], the apparent cause of delayed onset is still under debate. However, the earlier unawareness of myocarditis might be the reason. From 30 cases of ICI inhibitor-related cardiotoxicity, more than half (18 cases) of cardiac events were linked to anti-PD-1/PDL-1 inhibitors from the time of treatment. The median period to cardiac toxicity presentation was 65 days and mostly after the first and third cycle (nearly within three cycles). In another study of 35 cases with anti-PD-1/PDL-1 inhibitors (monotherapy, 23 cases; combined with anti-CLT-4, 12 cases), in which 76% of heart- related toxicity occurred within the first six weeks of treatment [59]. Presentation of ICI-associated myocarditis can vary from non-specific to fulminant. Patients can experience exhaustion, dyspnea, orthopnea, myalgia, palpitation, chest pain, edema of the lower extremity, lightheadedness, syncope, or shifts in mental status [64]. Severe cases of cardiogenic shock or cardiac arrest can occur. In a large retrospective analysis, myocarditis and pericarditis have been observed more in anti-PD- or anti-PDL-1 monoclonal antibodies relative to those treated with anti-CTLA-4 monotherapy, and this may be due to the increased use of anti-PD-1 or anti-PDL-1 regimens over anti-CTLA-4 monotherapy [4]. Dyspnea and palpitation were the most frequent clinical symptoms, and left ventricular systolic disturbance was identified in a majority of patients (79%) [34]. However, atrial fibrillation, ventricular arrhythmia, and conduction abnormalities were also found. Patients with myocarditis related to PD-1/PDL-1 inhibitors may have pre-existing or concomitant non-cardiac irAEs [61]. Given that there is no significant difference between all forms of immune checkpoint inhibitors as monotherapy or combination inhibitors, anti-PD-1 and anti-PDL-1 antibodies have almost similar incidences of concomitant diseases [4], which are mostly myositis and myasthenia gravis [61]. In addition, patients with underlying autoimmune conditions, pre-existing cardiovascular disease, or diabetes mellitus may have a higher risk [31], [44] of developing any related cardiotoxicity symptoms or concomitant diseases; therefore, immediate examination with referral to the cardiology unit should be initiated [46]. Although anti-PD-1/PDL-1 antibodies have been found to cause cardiotoxicity, a case series reported that anti-PDL-1 inhibitors (atezolizumab, durvalumab, avelumab) result in less immune-mediated myocarditis than anti-PD-1 drugs (Table 2). The FDA has identified PD-1 inhibitor (cemiplimab) as a cardiotoxic medication. However, there is no evidence showing its cardiac toxicity so far.

**Table 2:** Recent published series on anti-PDL-2 inhibitors-related cardiotoxicity.

Study*	Old (sex)	Type of cancer	Anti-PD-L1 type	Symptoms/Cardiotoxicity		Outcome
(Mahmood et al., 2018 [43]	75,F	Metastatic endometrial cancer	Durvalumab 1500 mg + tremelimumab 75 mg	Difficulty ambulating, dyspnea / Myocarditis, HF,CHB	Started on IV Methylprednisolone 1 mg/kg to 20 mg/kg on day 2, mycophenolate mofetil 1000 mg oral twice daily	Symptoms improve
(Altan et al., 2019) [60]	72/M	Lung cancer	Anti-PD-L1	Dyspnea, hypotension hypoxia / Pericarditis	N/A	Death
Altan et al., 2019) [65]	57/F	Lung cancer	Anti-PD-L1	Dyspnea, orthopnea, bilateral lower edema / Cardiac tamponade	N/A	No additional toxicity after reintroduction
(Liu et al., 2019) [66]	61/F	Lung cancer	Atezolizumab and nivolumab	Dyspnea, fatigue / Myocarditis		Deterioration
(Berner et al., 2018 [67]	69/M	Renal cell carcinoma	Avelumab and Axitinib	Fatigue, constipation hypertension / Cardiac arrest	Reduction of axitinib, amlodipine	Death
(Li J et al., 2019) [68]		Non-small cell lung cancer	Atezolizumab	Left ventricular dyfunction		Death

* Reported as case reports.

## Management

### Diagnosis

There are no specific diagnostic guidelines for this relatively newly rising cardiotoxicity, but increasing, understanding, and practicing will hopefully evolve [69]. Clinical diagnosis by detailed history and physical examination in combination with multimodality investigation tools such as cardiac biomarkers and imaging can help in the evaluation of patients at risk for cardiotoxicity related to PD-1/PDL-1 administration. Individuals with suspected high-risk cardiac involvement during treatment (history of cardiac disease, lung cancer, combined immunotherapy, or cardiotoxic chemotherapy) should be evaluated by a cardiologist or relevant qualified cardio-oncologist if present [70].

In systemic conditions, autoimmune conditions such as sarcoidosis, polymyositis, rheumatoid arthritis, and systemic lupus erythematosus affect cardiac tissues [71]. Thus, they lead to many heart disorders, including heart failure, arrhythmias, and ventricular dysfunction, which can be exacerbated by subclinical myocarditis, which cannot be detected by a single diagnostic tool of potential value. With PD-1/PDL-1 administration, clinicians should pay attention and use more diagnostic methods to confirm suspected heart problems with related causes, including non-inflammatory issues such as dilated cardiomyopathy and Takotsubo syndrome [30].

**Fig. 2:**
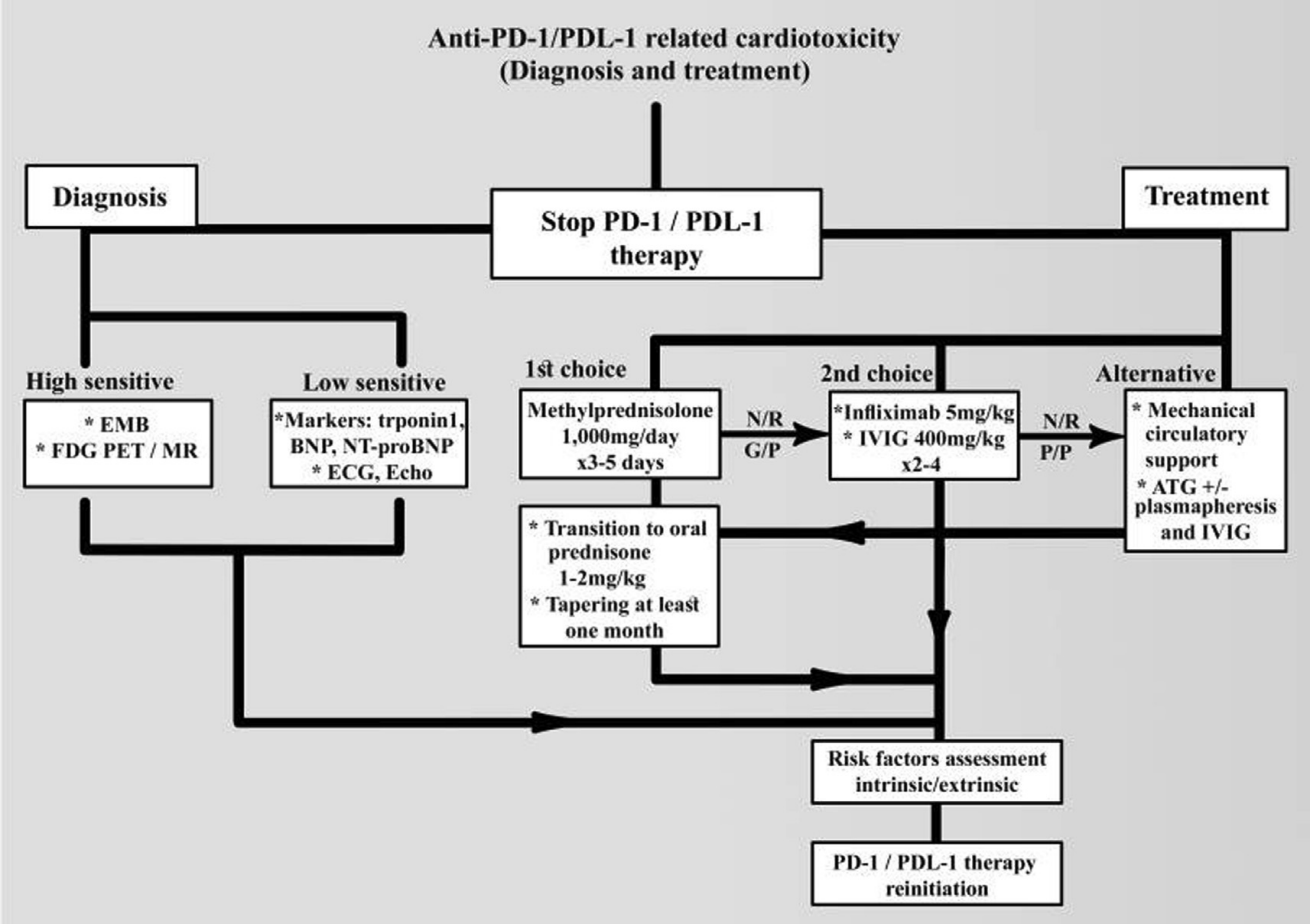
Diagnostic and treatment outlines for PD-1/PDL-1 drugs-related cardiotoxicity complications. FDG/PET: fluorodeoxyglucose/positron emission tomography, EMB: endomyocardial biopsy, BNP: brain natriuretic peptide, NT-proBNP: N-terminal pro-brain natriuretic peptide - N/R: no response. G/P: good performance. P/P: poor performance.

In addition to the early evaluation of the standard diagnosis of anti-PD-1/PDL-1-associated cardiotoxicity (e.g., electrocardiogram echocardiography), a more detailed review of practical techniques is given in the relevant literature [72], [73], [74]. First, EMB is the gold standard for diagnosing PD-1/PDL-1-induced myocarditis with lymphocyte and macrophage infiltration of myocardial fibrosis tissues. Due to severe complications and its invasive nature with associated risks, it is reserved only for those who are refractory to induction treatment and who have doubts about the diagnosis. Second, FDG PET/MRI has less sensitive results as it is diagnosing myocarditis (detecting myocardial edema only in 5 of 15 cases (33%) and late gadolinium enhancement that indicates fibrosis replacement and acute myocarditis has been reported within just 3 out of 13 cases (23%) [47]. However, recent retrospective and case-based observational studies have suggested complementary and incremental criteria for using FDG PET/MRI for myocarditis in contrast to PET/CT or MRI alone [75]. Third, in terms of markers, cardiac troponin elevation has been reported in 46% of myocarditis cases in one study; thus, it is not a sensitive biomarker [76]. The concentrations of brain natriuretic peptide (BNP) or N-terminal pro-BNP are increased by 100% of immunotherapy-related cardiac problems with outstanding negative predictive value concerning left ventricular dysfunction and heart failure. Although BNP and NT-pro-BNP are non-specific cardiac dysfunction biomarkers, we consider BNP to be a useful biomarker for screening. Broader studies have contributed to an effective way of ascertaining early cardiotoxicity related to PD-1/PDL-1 therapy. While electrocardiogram or coronary echocardiogram monitoring and screening are prescribed for pre-existing diseases in most cancer patients, minimal early data suggest no difference in the results between treatment and control groups before starting immunotherapy [46], [77]. Here, we briefly explain the best diagnostic tools for PD-1/PDL-1 monoclonal therapy Fig. 2.

#### Myocarditis

Myocarditis can be challenging to be clinically diagnosed. Thus, irAEs that are closely related to myocarditis, such as myositis, pulmonary edema, decompensated heart failure, ventricular arrhythmias, cardiac shock, and multi-organ failure, could lead to death and provide evidence of the myocarditis unequivocally. Myocarditis is marked by new left ventricular dysfunction, elevated cardiac serum troponin and BNP, and significant inflammation of the myocardial wall, as seen by FDG PET/MRI or EMB. Furthermore, the patients with EMB had histopathology with suspected associated immunotherapy, confirming lymphocytic penetration, with myocardial fibrosis at 56%. Immunohistochemistry can also be used as an adjunct to cell-specific markers (CD3, CD68, or human leukocyte antigens) and may increase diagnostic sensitivity [28], [71].

#### Pericarditis

This condition could appear as pressure or in conjunction with myocarditis (perimyocarditis), which might be exacerbated by pericardial effusion and extreme cardiac tamponade. It can be diagnosed by cardiac ultrasound; electrocardiogram adjustments that can present with irregular PR, widespread saddle-shaped ST acceleration, and atrial and ventricular arrhythmias; elevated troponin (only in perimyocarditis); and significant inflammation with cardiac MRI, ^18^F-FDG PET/CT, or FDG PET/MRI.

#### Arrhythmias

Arrhythmias generally present as atrial or ventricular arrhythmias (tachycardia or fibrillation may occur in various situations, such as poor-state cancer patients and not as in almost cardiotoxic events, but may also occur with PD-1/PDL-1 inhibitors administration). Concomitant myocarditis may present with any kind of arrhythmias [46], [61]. It is a conduction disease that might be associated with coexisting myocarditis or atrioventricular block. Atrioventricular conduction dysfunction is evidenced by various degrees of heart block, bradycardia, or sudden cardiac death resulting from total heart block. It is diagnosed by using an electrocardiogram or Holter testing. The usual manifestation in the electrocardiogram shows slowly prolonged PR interval, QRS axis deviation, bundle branch block, or second-degree or complete heart block.

#### Non-myocarditis cardiac dysfunction

In some situations, anti-PD-1/PDL-1 drugs lead to left cardiac dysfunction without evidence of myocarditis and present as a functional impairment called non-inflammatory left ventricular dysfunction, including dilated cardiomyopathy, Takotsubo syndrome, and unobstructed coronary arteries with high BNP and long-QT syndrome [78], [79]. Non-inflammatory left ventricular dysfunction mostly depends on the exclusion of active myocarditis that is tested by cardiac MRI, ^18^F-FDG PET/CT, or EMB.

#### Myocardial infarction MI

MI usually presents with chest pain, abnormal ST electrocardiogram ischemic changes (elevation or depression), T-wave inversion, a rise of cardiac troponin, and structural wall abnormalities in new echocardiogram or cardiac MRI. Furthermore, coronary vasospasm with ST-elevation has been reported and could exist secondary to PDL-1 inhibitor treatment [80]. Although elevated troponin makes the difference in acute coronary syndrome, whether troponin elevation is associated with increased rupture of atherosclerotic plaque, coronary vacuities caused by PD-1/PDL-1 antibodies, or focal myocarditis that is misdiagnosed as MI remains unknown. The highly sensitive device is coronary angiography, and while a suspect occlusion or extreme stenosis is observed, a percutaneous coronary intervention should be indicated.

#### Vasculitis

Patients with systemic inflammatory disorders that negatively affect vessels (rheumatoid arthritis, lupus erythematosus, psoriatic arthritis, systemic sclerosis, vasculitis, polymyositis) may also have subclinical myocarditis. As a consequence, the usage of anti-PD-1/PDL-1 treatments in patients with pre-existing autoimmune disorders should be directed by clinical practice [81]. In one study, vasculitis was reported 55 days after immunotherapy administration with a relatively lower mortality rate (6%) [82]. Vasculitis cases are usually resolved with discontinuation of anti-PD-1/PL-1 inhibitors and administration of steroids, and there was no mortality among these patients [30]. However, a biopsy may be recommended for the major complications of vasculitis and resistant cases.

## Treatment

Usually, medicinal or surgical interventions are the first-line treatment of heart problems, which have to be performed in accordance with the guidelines of standard cardiology societies to cope with emerging cases of cardiac toxicity. In general, conventional drugs for pre-existing heart diseases, heart attacks, and heart failure have been used for treatment. In the treatment of acute heart failure and pulmonary edema, angiotensin-converting enzyme inhibitors or intravenous nitrates and diuretics should be administrated. Support and advanced mechanical support include inotropic and ventricular tachyarrhythmia antagonists or amiodarone with effective cardioversion or defibrillation for hemodynamically unstable ventricular tachycardia and ventricular fibrillation. Surgical intervention is an aggressive alternative; however, it may be useful for treating blockages and heart problems for which medications may not be successful, especially in advanced heart disease stages. In individuals with arrhythmia, a portable left ventricular assist device or automated devices help to monitor heartbeat and could be another choice for total heart obstruction and heart transplants. Cases with massive pericardial effusions of cardiac tamponade and pericardiocentesis have to be treated early. In patients under PD-1/PDL-1 therapy, myocarditis associated with a high risk of fatality, especially in pre-existing cardiovascular or other cardiotoxic conditions and treatment, should be recommended early after PD-1/PDL-1 therapy [20]. After the diagnosis of myocarditis, left ventricular dysfunction (dilated cardiomyopathy or Takotsubo syndrome), arrhythmia, or MI, the discontinuation of PD-1/PDL-1 inhibitors and the start of corticosteroids have to be planned. The time of PD-1/PDL-1 treatment discontinuation or corticosteroid dose adjusting should be based on the grades of cardiac toxicity and manifestation. The decision of permanent discontinuation of steroids have to be strictly decided and as anti-PD-1/ PDL-1 inhibitors have a long plasma half-life and stopping treatment will not immediately reverse the pharmacodynamics of therapy and requires comprehensive discussion between the oncologist and cardiooncologist and safety as the recurrence and worse will be devastating [44] and EMB results may be considered [83]. Furthermore, attention should be paid to patients with remarkable existing or pre-existing risk factors of adverse synergetic cardiac effects. In those with pre-existing systemic autoimmune disorders (rheumatoid arthritis, lupus erythematosus, psoriatic arthritis, systemic sclerosis, vasculitis, polymyositis), anti-PD-1 inhibitor administration causes distinct mild and moderate forms of myocarditis [81]. According to the severity, cardiac symptoms due to immunotherapy, and grade 3–4 toxicity, PD-1/PDL-1 immunotherapy must be discontinued, and IV corticoids should be started. Then, the IV corticoids should be withdrawn gradually and replaced with oral steroid drugs for at least for one month. From the etiological driver of myocarditis, the indication of early high glucocorticoids is still the cornerstone. In most anti-PD-1/PDL-1 immune-related cardiac toxicities, a high dose of IV corticosteroids (methylprednisolone) is given, followed by an oral steroid taper at 1–2 mg/kg. Mahmood et al. found that the initial dose of 1000 mg relived most of the cardiac events [46]. However, other patients still have serious arrhythmias and heart failure [13], [31], and guidelines for further specific immune-related anti-PD-1/PDL-1 treatment are highly recommended. In another series of left ventricular dysfunction, although most of the cardiac complications were effectively treated by a high dose of corticosteroids and take long term recovery in eight (67%) of the 12 cases [31], [50]. Of note, corticosteroid use is associated with curative effects or no return of cardiotoxicity seen in patients who underwent ICI re-administration [84]. In patients that cannot tolerate corticosteroids, infliximab and mycophenolate mofetil and plasma exchange could be regarded as second-line therapies [28], [85]. In concurrent anti-PD-1 receptors with refractory toxicity (nivolumab) under steroid-refractory myocarditis, patients showed significant improvement in biochemical (troponin) and clinical cardiac manifestations after infusions of two to three doses of infliximab at 5 mg/kg (a chimeric IgG1 monoclonal antibody that blocks tumor necrosis factor-α, a pro-inflammatory cytokine) [84], [85]. Nevertheless, one center found that infliximab showed a reasonable success rate in patients with heart failure and suggested that the drug should be used with caution [86], [87]. In patients with poor performance or failure to respond to high-dose steroids, antithymocyte globulin ATG or intravenous immunoglobulin should be considered together. In the case of cardiogenic shock, advanced heart failure specialist in using immunosuppression, mechanical circulatory support, and inotropic treatments is highly recommended [88], [89], [90]. ATG is polyclonal and inotropic. Its indications primarily pertain to allograft rejection and aplastic anemia [91]. Tay et al. had also confirmed that ATG leads to a reduction in T-cell hyperactivation and lymphocytic infiltration inactivation, thereby controlling myocarditis and its negative impacts [88]. Several studies have been conducted on the use of ATG for immune-related cardiotoxicity [92], [93]. In one individual with nivolumab-related cardiac abnormal conduction, ATG was given, and the patient restored from total heart block to sinus rhythm with second-degree heart block with intermittently dropped beats. One case of a cardiogenic shock from extreme myocarditis due to anti-PD-1/PDL-1 treatment has been treated with a single dose of ATG, resulting in a substantial rise in blood pressure [84]. Although there are several limitations in using therapeutic plasma exchange-associated drug removal, unstable patients should be advised to take plasmapheresis treatment [94]. One patient with lung cancer was placed in the ICU after three doses of nivolumab, and plasmapheresis was conducted to decrease the level of nivolumab from 45.1 μg/ml (asterisk) before plasmapheresis to 5.6 μg/ml after the first plasmapheresis session. If abnormal biochemical and clinical instability further increased, intravenous abatacept (a CTLA-4 agonist) at a dose of 500 mg every two weeks for a total of five doses has to be administered. Abatacept may lead to rapid inactivation of normal immune response and thus reverse anti-PD-1/PDL-1 inhibitor pathways; however, further precise evaluation of this drug is highly recommended [95]. Atezolizumab is another monoclonal that binds to CD52, a protein present on the surface of most immune cells (not stem cells). It works by complement-mediated inhibition that leads to the resolution of cardiac toxicity by cytolytic enhancement of immunosuppression [96] Fig. 2.

## Conclusion

Cancer immunotherapy has notably evolved in clinical practice, and awareness of adverse events, especially lethal cardiotoxicity, has increased. However, there are no specific guidelines regarding the best management plan of PD-1/PDL-1 immunotherapeutic drugs. Thus, more studies are needed to develop guidelines regarding the use of PD-1/PDL-1 immunotherapeutic drugs. However, it gives the simple outlines of applying for clinicians, further awareness, and comprehensive preclinical and clinical testing of cardiac toxicity methods highly advised for future suspected overuse in the oncology centers.

## Abbreviations

FDA, Food and Drug Administration; NSCLC, Non-small cell lung cancer; PD, Programmed cell death; PDL, Programmed cell death ligand; D.C.s, Dendritic cells; trAEs, Treatment-related adverse events; irAEs, Immune-related effects; ICI, Immune checkpoint inhibitors; EMB, Endomyocardial biopsy; MI, Myocardial infarction; Raf, Rapidly Accelerated Fibrosarcoma kinase; MEK, Mitogen-Activated protein Kinase inhibitors; VEGF, Vascular Endothelial Growth Factor.

## Funding

None declared.

## Compliance with Ethics Requirements

This article does not contain any studies with human or animal subjects.

## CRediT authorship contribution statement

**Mohammed Safi:** Conceptualization, Validation, Formal analysis, Investigation, Writing - original draft, Project administration. **Yun-long Xia:** Supervision. **Hyat Ahmed:** Formal analysis, Investigation, Writing - original draft. **Mahmoud Al-Azab:** Investigation, Writing - original draft. **Xiu Shan:** Validation. **Mohammed Al-radhi:** Visualization. **Abdullah Al-danakh:** . **Abdullah Shopit:** . **Jiwei Liu:** Conceptualization, Formal analysis, Supervision, Project administration.

## Declaration of Competing Interest

The authors declare that they have no known competing financial interests or personal relationships that could have appeared to influence the work reported in this paper.
